# Short Mediterranean diet screener detects risk of prediabetes in Taiwan, a cross-sectional study

**DOI:** 10.1038/s41598-023-28573-5

**Published:** 2023-01-21

**Authors:** Yi-Cheng Hou, Jui-Yu Li, Jiann-Hwa Chen, Jong-Kai Hsiao, Jing-Hui Wu

**Affiliations:** 1 Department of Nutrition, Taipei Tzu Chi Hospital, Buddhist Tzu Chi Medical Foundation, No. 289, Jianguo Rd., Xindian Dist., New Taipei City, 231 Taiwan; 2 Division of Gastroenterology and Hepatology, Department of Internal Medicine, Taipei Tzu Chi Hospital, Buddhist Tzu Chi Medical Foundation, New Taipei City, Taiwan; 3 Department of Medical Imaging, Taipei Tzu Chi Hospital, Buddhist Tzu Chi Medical Foundation, New Taipei city, Taiwan; 4 School of Medicine, Tzu Chi University, Hualien, Taiwan

**Keywords:** Nutrition, Public health

## Abstract

We aimed to determine whether the 14-item Mediterranean diet adherence screener (MEDAS) is suitable in Taiwan and associate the MEDAS score with the risk of prediabetes. In this cross-sectional study 346 patients were recruited between 2014 and 2019 at Taipei Tzu Chi Hospital. The MEDAS score was obtained with a 14-item MEDAS used in the PREDIMED trial. The blood glucose level is measured by fasting glucose and HbA_1c_. The results of the screener were analyzed for internal consistency and compared with the blood glucose level using multivariate regression models. The MEDAS score was significantly (p = 0.001) and inversely associated with both measures of blood glucose level. Adjusted data (95% CI) showed that each additional point in the MEDAS score decreases the risk of prediabetes with abnormal fasting glucose (> 100 mg/dL) level by 60% and the risk of prediabetes with abnormal HbA_1c_ (> 5.7%) by 22.4%. Consuming at least 3 servings of legumes each week was significantly (p = 0.007) related to a lower risk of prediabetes under logistic regression. A higher score on the 14-item MEDAS screener was significantly associated with a lower risk of prediabetes.

## Introduction

Prediabetes is considered a high-risk state of type 2 diabetes and a reversible condition with lifestyle changes^1^. American Diabetes Association (ADA) defined prediabetes using three measures: fasting glucose 100–125 mg/dL, glycated hemoglobin 5.7–6.4%, or glucose tolerance test resulting in 140–199 mg/dL two hours after ingesting a standardized 75 g glucose solution^2^. The Nutrition and Health Survey in Taiwan 2013–2016 showed that 29.6% of the adults in Taiwan have prediabetes defined by impaired fasting glucose alone^3^. Patients with prediabetes are encouraged to adopt a health-promoting lifestyle that includes dietary change, exercise, and weight control while maintaining regular check-ups^4–7^.

Knowledge and awareness of the warning signs of chronic diseases may encourage people meeting disease criteria to initiate early treatments^8^. According to Protection Motivation Theory (PMT) which was proposed as a general concept for predicting health behaviors and health-related interventions^9^. In conclusion, it may appropriately designed healthy eating and/or exercise interventions and effectively prevent chronic diseases^10–12^.

The Mediterranean diet is recommended by ADA and supported by multiple studies as a dietary pattern that may reduce the risk of type 2 diabetes^13–16^. The traditional Mediterranean diet is characterized by high consumption of vegetables, legumes, grains, fruits, nuts, and olive oil; moderate consumption of fish and wine; low consumption of red and processed meat and whole-fat dairy products^17^. A 14-item questionnaire was developed as a brief Mediterranean diet adherence screener (MEDAS), instead of a full-length food frequency questionnaire, in the PREDIMED trial to evaluate the degree of adherence to the Mediterranean diet^18^. The MEDAS score has been used in studies regarding metabolic syndromes and prediabetes^7,19,20^. The MEDAS score had been targeted on different groups like older Spanish, women with BRCA1/2 mutation in Germany or people at high cardiovascular risk in the UK^21–23^. It also been translated into Brazilian Portuguese language or used in telephone-administered version^24,25^. Numerous studies validated the MEDAS score used in very different regions, target groups, and methods.

Prediabetes is the critical stage for diabetes prevention. The MEDAS items are concrete yet simple pointers useful in self-evaluations and dietary interventions. This study investigated the association between the degree of adherence to the Mediterranean diet and blood glucose level in prediabetes patients and healthy controls recruited in Taiwan. The items in MEDAS were individually analyzed to clarify the effectiveness of MEDAS in Taiwan and prompted possible modifications for future use. This was the first study in Taiwan to use a Mediterranean diet adherence score to investigate the connection between Mediterranean diet and prediabetes. As part of our study, dietary records were collected to cross check the MEDAS responses and reliability of the MEDAS was analyzed by evaluating internal consistency of the questionnaire.

## Materials and methods

### Participants

In this cross-sectional study, 346 patients between 20 and 65 years of age were recruited between 2014 and 2019 at the Taipei Tzu Chi Hospital through the Division of Family Medicine, the Division of Metabolism, and the Healthcare Center. The exclusion criteria were all kinds of diabetes and had diabetes history, psychiatric or neurological illness, surgery (within 10 years), hypertension, dyslipidemia, smoking, thyroid dysfunction, asthma, adrenal dysfunction, parathyroid dysfunction, cancer, pregnancy, or renal dialysis. We especially excluded patients with hypertension and dyslipidemia to avoid the confounding factors by avoiding the potential confounding role of these factors. On the days of recruiting, the medical records of patients were screened to remove patients satisfying the exclusion criteria. The recruiter would explain our study to patients that had their blood tested on that day. Since most patients were going to the hospital for a reason, it took a long time to recruit enough participants for both the healthy group and the prediabetes group.

The original estimate was 219 participants. Due to some participants missing one of the fasting glucose or the glycated hemoglobin, the recruiting continued until a safe number of participants was reached.

### Nutrition assessment

Anthropometric data, 24-h dietary recall, 3-day food record, and the 14-item Mediterranean diet adherence screener were collected from the participants by the recruiter and a dietitian once during the whole process. The recruiter was in charge of collecting data while the dietitian was in charge of explaining serving sizes and questionnaire instructions. Blood glucose data were collected from the subjects via their blood test. The hospital laboratory used point-of-care testing to assay fasting glucose and enzyme immunoassay to assay glycated hemoglobin. The blood glucose level is measured by fasting glucose (mg/dL) and glycated hemoglobin (%).

After removing cases of incomplete or missing data, a total of 335 subjects completed the MEDAS. Among them, 242 subjects (72.2%) had fasting glucose measured, and 196 subjects (58.5%) had glycated hemoglobin measured. Although the MEDAS had been validated in PREDIMED trial in Europe not in Taiwan, we had adjusted the portion size of the food questionnaire. Additionally, the food referred in the questionnaire were not significant difference between Taiwan and Europe. The reliability and validity coefficient of questionnaire is higher than 0.8.

### Food record and physical activity questionnaire

The 24-h dietary recall, physical activity questionnaire, and the 3-day food record provided intake data of nutrients. The 24-h dietary recall and the physical activity questionnaire was completed right after recruitment by asking the participants in person. Physical activity data collected consider the amount and intensity of activities. The 3-day food record was given to the participants to be completed and mailed back. Dietary records were inputted into the E-Kitchen software in order to obtain nutrients data. The MEDAS is translated into Chinese and administered by dietitians. Each item is assigned either 0 or 1 point based on their answer. The 14 items are: (1) use of olive oil as main culinary fat, (2) olive oil > 4 tablespoons/day, (3) vegetables ≥ 2 servings/day, (4) fruits ≥ 3 servings/day, (5) red/processed meats < 1 serving/day, (6) butter/cream/margarine < 1 serving/day, (7) Sweet beverages and carbonated drinks < 1 glass/day, (8) wine ≥ 7 glasses/week, (9) legumes ≥ 3 servings/week, (10) fish/seafood ≥ 3 servings/week, (11) commercial sweets and confectionery ≤ 2 servings/week, (12) tree nuts ≥ 3 servings/week, (13) poultry more than red meats, (14) use of sofrito sauce ≥ 2/week. The serving sizes are explained to the subjects based on common measuring units.

### Statistical analysis

The statistical analysis methods include one-way analysis of variables, Chi-square test, Pearson correlation coefficient, and logistic regression. A linear multiple regression with variable error and incomplete data was utilized to estimate the minimum required sample size. The correlation and validation for the 3-day food diary and the MEDAS have been tested and the results can be seen in the supplementary section^26^. The reliability of the MEDAS result measures the internal consistency of the items. The MEDAS is a dichotomy-type questionnaire, therefore the Cronbach’s alpha that resulted from the analysis will be skewed toward the least consistent item. The four least consistent items in our results are (5) red/processed meats < 1 serving/day, (11) commercial sweets and confectionery ≤ 2 servings/week, (13) poultry more than red meats, and (6) butter/cream/margarine < 1 serving/day. The Cronbach’s alpha reached 0.707, good reliability, if the items (5), (11), (13), and (6) were removed. Due to the inconsistent items, the 14-item MEDAS score would have a lower reliability compared to a modified 10-item screener. After careful consideration, we decided to keep the MEDAS unmodified in this study.

### Ethics statement

The study was approved by the Institutional Review Board of Taipei Tzu Chi Hospital, Buddhist Tzu Chi Medical Foundation (approval number 02-XD14-043), and followed the ethical standards of the Declaration of Helsinki for Medical Research in Humans (2013) and the Oviedo Con-vention (1997). Written informed consent was obtained from all subjects and/or their legal guardian(s).

## Results

Subjects were split into three groups based on their MEDAS score: low adherence group has scores between 0 and 3 (n = 54, 16.1%); medium adherence group has scores between 4 and 6 (n = 188, 56.1%); good adherence group has scores at least 7 (n = 93, 27.8%). The original grouping was done by quintiles. The scores of the quintile groups were 0–3, 4, 5, 6, and 7–14. All subjects scored less than 12 points. We chose to group scores 4–6 together for the ease of presenting tables. Table 1 showed the characteristics of each group. The amount of carbohydrate, folic acid, and docosahexaenoic acid consumed were significantly related to the MEDAS score. There was also a correlation between simple sugar intake and the MEDAS score if we omit the low adherence group. Physical activity data collected consider the amount and intensity of activities. Intensity of physical activity was significantly related to the MEDAS score, where the medium and good adherence groups preferred low intensity physical activities.

**Table 1:** Characteristics of participants and blood glucose level by MEDAS score.

	MEDAS score			p^abc^
	Low 0–3	Medium 4–6	Good 7–14	
	n = 54 (16.1)	n = 188 (56.1)	n = 93 (27.8)	
Sex				0.371^b^
Female	37 (68.5)	109 (58.0)	55 (59.1)	
Male	17 (31.5)	79 (42.0)	38 (40.9)	
Age (years)	55.07 ± 10.65	53.78 ± 11.08	51.75 ± 10.02	0.156^a^
BMI (kg/m^2^)	24.02 ± 3.91	24.22 ± 3.21	23.19 ± 3.44	0.060^a^
Underweight (< 18.5 kg/m^2^)	5 (9.4)	0 (0.0)	4 (4.3)	< 0.001^b^
Normal (18.5–23.9 kg/m^2^)	20 (37.7)	101 (53.7)	59 (64.1)	
Overweight (24.0–26.9 kg/m^2^)	17 (32.1)	54 (28.7)	13 (14.1)	
Obese (> 27.0 kg/m^2^)	11 (20.8)	33 (17.6)	16 (17.4)	
Energy (kcal)	1573.03 ± 549.50	1432.39 ± 545.54	1438.02 ± 480.18	0.212^a^
Protein (g)	56.44 ± 21.08	53.26 ± 24.18	56.11 ± 23.36	0.516^a^
Fat (g)	56.31 ± 34.53	63.79 ± 56.07	66.15 ± 85.73	0.651^a^
Carbohydrate (g)	230.70 ± 120.82	260.73 ± 194.93	334.68 ± 304.88	0.009^a^
Fiber (g)	3.93 ± 2.71	3.63 ± 3.90	25.16 ± 164.72	0.131^a^
Cholesterol (mg)	260.86 ± 218.03	209.15 ± 192.06	258.18 ± 184.55	0.068^a^
Simple sugar (g)	4.00 ± 7.22	2.87 ± 6.59	12.24 ± 36.16	0.001^a^
Vitamin E (mg)	4.24 ± 2.90	4.72 ± 3.51	13.26 ± 66.35	0.131^a^
Vitamin B12 (μg)	0.94 ± 0.71	0.76 ± 0.59	10.71 ± 95.20	0.272^a^
Folic acid (μg)	0.08 ± 0.56	2.42 ± 11.66	9.35 ± 30.37	0.003^a^
Vitamin C (mg)	96.43 ± 90.22	104.67 ± 95.35	127.36 ± 99.37	0.097^a^
Eicosapentaenoic acid (mg)	1.02 ± 3.79	31.61 ± 174.18	64.26 ± 215.02	0.093^a^
Docosahexaenoic acid (mg)	14.81 ± 51.29	74.90 ± 317.87	167.81 ± 357.16	0.008^a^
Physical activity				
Low intensity	14 (25.9)	57 (30.3)	56 (60.2)	< 0.001^b^
Moderate intensity	40 (74.1)	131 (69.7)	37 (39.8)	
Blood glucose level				
Fasting glucose (mg/dL)	106.51 ± 11.20	106.44 ± 20.56	91.19 ± 8.18	< 0.001^c^
Normal (< 100)	7 (14.3)	47 (31.3)	41 (95.3)	
Abnormal (100–125)	42 (85.7)	103 (68.7)	2 (4.7)	
Glycated hemoglobin (%)	5.88 ± 0.46	5.78 ± 0.61	5.43 ± 0.40	0.001^c^
Normal (< 5.7)	12 (32.4)	49 (40.8)	28 (71.8)	
Abnormal (5.7–6.4)	25 (67.6)	71 (59.2)	11 (28.2)	

One-way analysis of ^a^variables (continuous variables) or ^b^Chi-squared tests (categorical variables). ^c^Crude linear regression model.

Data are presented as the mean ± standard deviation for continuous variables and n (%) for categorical variables. *MEDAS* Mediterranean diet adherence screener, *BMI* Body Mass Index.

Using the ADA definition, abnormal fasting glucose referred to 100–125 mg/dL and abnormal glycated hemoglobin referred to 5.7–6.4% in blood glucose level. Table 1 showed the number of subjects in each adherence group according to the glucose level. A negative correlation was found between the MEDAS score and the fasting glucose level among 242 subjects. A negative correlation was found between the MEDAS score and glycated hemoglobin level among 196 subjects. Table 2 analyzed the odds ratio in three different logistic regression models. In the unadjusted model, the risk of abnormal fasting glucose went down by 58.3% for each additional point in the MEDAS score. In the unadjusted model, the risk of abnormal glycated hemoglobin went down by 23.9% for each additional point in the MEDAS score. In the model that is fully adjusted by characteristics in Table 1, the risk of abnormal fasting glucose and glycated hemoglobin went down by 60.0% and 22.4%, respectively, for each additional point in the MEDAS score.

**Table 2:** Multivariable-adjusted odds ratios (95% confidence intervals) from logistic regression for abnormal blood glucose level by MEDAS score.

Outcomes/model	MEDAS score						
	0–3	4–6		7–12		For + 1 point	
Fasting glucose (mg/dL)	n = 49	n = 150		n = 43			
Model 1	(Ref.)	0.365 (0.153–0.873)	0.023	0.008 (0.002–0.041)	< 0.001	0.417 (0.327–0.531)	< 0.001
Model 2	(Ref.)	0.245 (0.088–0.687)	0.008	0.004 (0.001–0.026)	< 0.001	0.381 (0.286–0.509)	< 0.001
Model 3	(Ref.)	0.261 (0.092–0.738)	0.011	0.005 (0.001–0.029)	< 0.001	0.386 (0.289–0.515)	< 0.001
Glycated hemoglobin (%)	n = 37	n = 120		n = 39			
Model 1	(Ref.)	0.696 (0.319–1.515)	0.361	0.189 (0.071–0.502)	0.001	0.761 (0.654–0.885)	< 0.001
Model 2	(Ref.)	0.601 (0.245–1.478)	0.268	0.223 (0.075–0.667)	0.007	0.802 (0.683–0.942)	0.007
Model 3	(Ref.)	0.615 (0.249–1.518)	0.291	0.223 (0.074–0.672)	0.008	0.803 (0.683–0.944)	0.008

*Model 1* unadjusted, *Model 2* adjusted for gender, age, BMI, *Model 3* additionally adjusted for nutrient intake, *MEDAS* Mediterranean diet adherence screener.

The percentage of positive answers from 335 subjects for each item in the MEDAS is shown in Table 3. The odds ratios for abnormal fasting glucose and abnormal glycated hemoglobin corresponding to each item are computed from 242 and 196 subjects, respectively. Items (6) butter/cream/margarine < 1 serving/day, (7) Sweet beverages and carbonated drinks < 1 glass/day, and (5) red/processed meats < 1 serving/day are the top three items with 92.84%, 82.99%, and 77.01% positive, respectively. Items (8) wine ≥ 7 glasses/week, (2) olive oil > 4 tablespoons/day, and (10) fish/seafood ≥ 3 servings/week are the lowest three items with 6.27%, 8.66%, and 12.54% positive, respectively.

**Table 3:** Multivariable-adjusted odds ratios (95% confidence intervals) from logistic regression for abnormal blood glucose level according to the fulfillment of each item in the MEDAS.

14-item MEDAS	Number of positive case at recruitment (%)	Fasting glucose odds ratio (95% CI), p		Glycated hemoglobin odds ratio (95% CI), p	
(1) Use of olive oil as main culinary fat	87 (25.97)	0.028 (0.009–0.091)	< 0.001	0.520 (0.233–1.158)	0.110
(2) Olive oil > 4 tablespoons/day	29 (8.66)	0.040 (0.005–0.349)	0.004	0.659 (0.183–2.380)	0.525
(3) Vegetables ≥ 2 servings/day	68 (20.3)	0.439 (0.210–0.919)	0.029	0.521 (0.237–1.146)	0.105
(4) Fruits ≥ 3 servings/day	71 (21.19)	0.318 (0.155–0.654)	0.002	0.729 (0.331–1.608)	0.434
(5) Red/processed meats < 1/day	258 (77.01)	1.915 (0.931–3.942)	0.078	1.779 (0.815–3.885)	0.148
(6) Butter. cream. margarine < 1/day	311 (92.84)	1.457 (0.488–4.352)	0.500	5.129 (1.269–20.729)	0.022
(7) Sweet beverages and carbonated drinks < 1/day	278 (82.99)	0.178 (0.067–0.468)	< 0.001	0.656 (0.272–1.580)	0.347
(8) Wine glasses ≥ 7/week	21 (6.27)	0.348 (0.083–1.458)	0.149	0.376 (0.071–2.005)	0.252
(9) Legumes ≥ 3/week	121 (36.12)	0.012 (0.003–0.054)	< 0.001	0.342 (0.156–0.749)	0.007
(10) Fish/seafood ≥ 3/week	42 (12.54)	0.146 (0.048–0.442)	< 0.001	0.294 (0.089–0.971)	0.045
(11) Commercial sweets and confectionery ≤ 2/week	219 (65.37)	1.866 (1.007–3.457)	0.047	0.928 (0.466–1.847)	0.831
(12) Tree nuts ≥ 3/week	73 (21.79)	0.037 (0.011–0.118)	< 0.001	0.383 (0.167–0.882)	0.024
(13) Poultry more than red meats	120 (35.82)	0.772 (0.405–1.471)	0.431	0.479 (0.237–0.967)	0.040
(14) Use of sofrito sauce ≥ 2/week	134 (40)	0.073 (0.034–0.158)	< 0.001	0.448 (0.223–0.899)	0.024

Adjusted for gender, age, BMI. *MEDAS* Mediterranean diet adherence screener, *CI* confidence interval.

Logistic regression shows that items (1) use of olive oil as main culinary fat, (2) olive oil > 4 tablespoons/day, (3) vegetables ≥ 2 servings/day, (4) fruits ≥ 3 servings/day, (7) Sweet beverages and carbonated drinks < 1 glass/day, (9) legumes ≥ 3 servings/week, (10) fish/seafood ≥ 3 servings/week, (12) tree nuts ≥ 3 servings/week, (14) use of sofrito sauce ≥ 2/week are associated with lower risk of abnormal fasting glucose, but the item (11) commercial sweets and confectionery ≤ 2 servings/week is associated with higher risk of abnormal fasting glucose.

Logistic regression shows that items (9) legumes ≥ 3 servings/week, (10) fish/seafood ≥ 3 servings/week, (12) tree nuts ≥ 3 servings/week, (13) poultry more than red meats, (14) use of sofrito sauce ≥ 2/week are associated with lower risk of abnormal glycated hemoglobin, but the item (6) butter/cream/margarine < 1 serving/day is associated with higher risk of abnormal glycated hemoglobin.

## Discussion

In this study, we found that patients who had a higher Mediterranean diet score were low in fasting glucose or glycated hemoglobin which could be associated with a lower risk of prediabetes. This further supported the claim that the Mediterranean diet reduces the risk of type 2 diabetes^13^. The inverse correlation between the MEDAS score and the risk of impaired fasting glucose was also shown in the literature^19,21^. To our knowledge, this was the first study in Taiwan that considers the MEDAS score as a possible association between Mediterranean diet and prediabetes. We chose to exclude diabetic subjects in this cross-sectional study to avoid confounding factors including diabetes medication and dietary intervention.

The degree of adherence to the Mediterranean diet was measured in several different methods in the literature^13,14,17,18,22^. The 14-item Mediterranean diet adherence screener developed by Martínez-González et al.^21^. was an easy-to-use brief survey that does not require a comprehensive food frequency questionnaire or estimated nutrient intake. Due to regional and cultural differences, usage of the MEDAS toward different target population required careful study and modification if necessary. It is also worth noting that adherence to the Mediterranean diet is only a small part of the ever-growing list of dietary quality indices that are developed for various purposes through history^23^. It is important to point out that our study was not designed to evaluate the effectiveness of a dietary intervention using the Mediterranean diet. Scoring a medium to high score on the MEDAS in this study would signify that the participant is conscious of the recommended healthy eating guideline in Taiwan and going above and beyond in the healthy categories. A perfect score on the MEDAS in Taiwan would mean consuming double or triple the recommended serving size on some food items. Every country has their own dietary guideline, typically written as minimal suggested amount. The MEDAS score measures how much participants go beyond the dietary guideline’s minimal suggestions.

In this study we found carbohydrate, folic acid, and docosahexaenoic acid intake to be associated with the MEDAS score. Despite the traditional Mediterranean diet having specific dietary recommendations, the MEDAS score did not reflect the adherence to the recommended nutrition intake. Carbohydrate, folic acid, and docosahexaenoic acid intake were associated with better blood glucose levels in some studies in particular scenarios^24,25,27^. We should also note that the percentage of total energy from carbohydrates remain in the normal range even in the good adherence group. In terms of the adherence to the Mediterranean diet, we found no literature on nutrition intakes with respect to the MEDAS. Studies on other measures confirm the complexity in this regard^28^. We identified subjects’ physical activity to address the limitations of MEDAS as a tool in dietary interventions. Subjects with more low intensity physical activities accounted for most medium or good MEDAS score group. This is not expected and prompts more detailed activity data in future studies.

The percentage of positive responses for each item at recruitment contains valuable information regarding the effectiveness of the MEDAS in Taiwan. The top three items (6) butter/cream/margarine < 1 serving/day, (7) sweet beverages and carbonated drinks < 1 glass/day, and (5) red/processed meats < 1 serving/day all refer to food items popular in the western diet. This may indicate that subjects prefer a diet which are not often used this kind of materials. The bottom two items (8) wine ≥ 7 glasses/week and (2) olive oil > 4 tablespoons/day come as no surprise. Despite wine and olive oil being advertised as healthy in Taiwan, the price still limits consumption. Item (8) is equivalent to daily consumption of wine, which is tough to achieve. Item (2) is a very high consumption of olive oil from the dietary guideline standpoint. The recommended oil consumption (not even olive oil) is 1 teaspoon per meal, far from the 12 teaspoons as stated in (2).

Almost all items in the MEDAS turned out to be associated with a lower risk of prediabetes. The overlapping items (9) legumes ≥ 3 servings/week, (10) fish/seafood ≥ 3 servings/week, (12) tree nuts ≥ 3 servings/week, (14) use of sofrito sauce ≥ 2/week agree with the recommendations toward a healthy diet^15,16,29^. The items that show the opposite association (6) butter/cream/margarine < 1 serving/day, (11) Commercial sweets and confectionery ≤ 2/week) are both dessert-related. Due to Asian desserts and western desserts being fundamentally different, there is a chance of confusion and clarifications should be added to the items. There is also the possibility of prediabetes subjects downplaying the amount of desserts they consume. Items (6) and (11) are also the less consistent items from the internal consistency analysis. We suspect the participants on average would not score on (6) and (11) but obese or prediabetes subjects report a lower consumption on purpose, resulting in low consistency.

The foods mentioned in the Mediterranean diet like olive oil, fruits, vegetables, fish/seafood are known as nutrient-dense, and include high concentrations of polyphenols, carotenoids, fiber, vitamins A, C, and E, polyunsaturated fat^30^. It has been known several benefits in terms of preventing cardiovascular disease, diabetes and also strengthen the immune system, reduce inflammation and oxidative stress^31^. We can also find out that the blood glucose level in 7–14 section become normal. It seems that in our study groups, people don’t need to be 100% met the diet to see the effect on blood glucose control and also indicate that the Mediterranean diet is effective in people who are prediabetes. Though there is other way to classified the subjects^18^, we still used different system based on the reason we found out.

This study has some limitations. In addition to the residual confounding, the recruitment of the subjects occurred at the Taipei Tzu Chi Hospital, a Buddhist hospital that promotes vegetarian diet. The hospital provides vegetarian food in their cafeteria and as meals to inpatients. The percentage of participants claiming to be vegetarian is 35%, a bit higher than the overall average ~ 13% in Taiwan. The blood glucose data of most subjects do not include both fasting glucose and glycated hemoglobin. The nutrition intake data collection did not include a comprehensive food frequency questionnaire. The cross-sectional study design cannot evaluate whether the MEDAS can be used as an effective tool in dietary interventions. Table 4 showed that according to the Cronbach’s α coefficient of the deleted items, this research deletes the items in order and updates the α coefficient as shown below. When undertaking the factor analysis results and only deleting the fifth question for reliability analysis, the Cronbach’s α coefficient 0.615 does not reach the standard of 0.70, after the 11th and 13th questions must be deleted, the Cronbach’s α coefficient of the Mediterranean Diet Scale is 0.707, which meets the standard of good reliability. At this time, there are 10 items left.

**Table 4:** The Cronbach’s α coefficient of the deleted items.

Process	Number of question	Cronbach’s α coefficient
Undertake factor analysis results	13	0.615
Delete Q11	12	0.667
Delete Q13	11	0.686
Delete Q6	10	0.707
Delete Q8	9	0.723
Delete Q7	8	0.726
Delete Q3	7	0.727
Delete Q4	6	0.730
Delete Q10	5	0.731

In conclusion, the MEDAS score is an effective indicator of prediabetes in Taiwan. A higher MEDAS score was significantly related to a lower risk of prediabetes from fasting glucose or glycated hemoglobin. The items (9) legumes ≥ 3 servings/week, (10) fish/seafood ≥ 3 servings/week, (12) tree nuts ≥ 3 servings/week, (14) use of sofrito sauce ≥ 2/week stood out as the main indicators. For the purpose of prediabetes prevention or management in Taiwan, recommending the main indicators as the initial intervention may be an effective strategy.

## Supplementary Information

Supplementary Information.

## Data Availability

The data sets used and/or analyzed during the current study available from the corresponding author on reasonable request.

## References

[1] Tabák AG, Herder C, Rathmann W, Brunner EJ, Kivimäki M (2012). Prediabetes: A high-risk state for diabetes development. Lancet.

[2] American Diabetes A (2011). Diagnosis and classification of diabetes mellitus. Diabetes Care.

[3] Nutrition and Health Survey in Taiwan (NAHSIT) [Internet] (Health Promotion Administration, Ministry of Health and Welfare, 2019).

[4] Chen MF, Hung SL, Chen SL (2017). Empowerment program for people with prediabetes: A randomized controlled trial. J. Nurs. Res..

[5] Luo Y, Paul SK, Zhou X, Chang C, Chen W, Guo X, Yang J, Ji L, Wang H (2017). Rationale, design, and baseline characteristics of Beijing Prediabetes Reversion Program: A randomized controlled clinical trial to evaluate the efficacy of lifestyle intervention and/or pioglitazone in reversion to normal glucose tolerance in prediabetes. J. Diabetes Res..

[6] Okada R, Tsushita K, Wakai K, Ishizaka Y, Kato K, Wada T, Watanabe K (2017). Lower risk of progression from prediabetes to diabetes with health checkup with lifestyle education: Japan Ningen Dock study. Nutr. Metab. Cardiovasc. Dis..

[7] Muscogiuri G, Barrea L, Di Somma C, Altieri B, Vecchiarini M, Orio F, Spinosa T, Colao A, Savastano S (2019). Patient empowerment and the Mediterranean diet as a possible tool to tackle prediabetes associated with overweight or obesity: A pilot study. Hormones.

[8] Yahia N, Brown C, Rapley M, Chung M (2014). Assessment of college students' awareness and knowledge about conditions relevant to metabolic syndrome. Diabetol. Metab. Syndr..

[9] Rogers RW (1975). A protection motivation theory of fear appeals and attitude change. J. Psychol..

[10] Vincent GE, Jay SM, Sargent C, Vandelanotte C, Ridgers ND, Ferguson SA (2017). Improving cardiometabolic health with diet, physical activity, and breaking up sitting: What about sleep?. Front. Physiol..

[11] Beaudry KM, Devries MC (2019). Nutritional strategies to combat type 2 diabetes in aging adults: The importance of protein. Front. Nutr..

[12] Meng Q, Lin MS, Tzeng IS (2020). Relationship between exercise and Alzheimer's disease: A narrative literature review. Front. Neurosci..

[13] Esposito K, Maiorino MI, Bellastella G, Chiodini P, Panagiotakos D, Giugliano D (2015). A journey into a Mediterranean diet and type 2 diabetes: A systematic review with meta-analyses. BMJ Open.

[14] Martinez-Lacoba R, Pardo-Garcia I, Amo-Saus E, Escribano-Sotos F (2018). Mediterranean diet and health outcomes: A systematic meta-review. Eur. J. Public Health.

[15] Management L (2019). Standards of medical care in diabetes-2019. Diabetes Care.

[16] Evert AB, Dennison M, Gardner CD, Garvey WT, Lau KHK, MacLeod J, Mitri J, Pereira RF, Rawlings K, Robinson S (2019). Nutrition therapy for adults with diabetes or prediabetes: A consensus report. Diabetes Care.

[17] Trichopoulou A, Martínez-González MA, Tong TY, Forouhi NG, Khandelwal S, Prabhakaran D, Mozaffarian D, de Lorgeril M (2014). Definitions and potential health benefits of the Mediterranean diet: Views from experts around the world. BMC Med..

[18] Martínez-González MA, García-Arellano A, Toledo E, Salas-Salvadó J, Buil-Cosiales P, Corella D, Covas MI, Schröder H, Arós F, Gómez-Gracia E (2012). A 14-item Mediterranean diet assessment tool and obesity indexes among high-risk subjects: The PREDIMED trial. PLoS One.

[19] Viscogliosi G, Cipriani E, Liguori ML, Marigliano B, Saliola M, Ettorre E, Andreozzi P (2013). Mediterranean dietary pattern adherence: Associations with prediabetes, metabolic syndrome, and related microinflammation. Metab. Syndr. Relat. Disord..

[20] McClure R, Villani A (2019). Greater adherence to a Mediterranean Diet is associated with better gait speed in older adults with type 2 diabetes mellitus. Clin. Nutr. ESPEN.

[21] Schröder H, Fitó M, Estruch R, Martínez-González MA, Corella D, Salas-Salvadó J, Lamuela-Raventós R, Ros E, Salaverría I, Fiol M (2011). A short screener is valid for assessing Mediterranean diet adherence among older Spanish men and women. J. Nutr..

[22] Sofi F, Macchi C, Abbate R, Gensini GF, Casini A (2014). Mediterranean diet and health status: An updated meta-analysis and a proposal for a literature-based adherence score. Public Health Nutr..

[23] Asghari G, Mirmiran P, Yuzbashian E, Azizi F (2017). A systematic review of diet quality indices in relation to obesity. Br. J. Nutr..

[24] Gower BA, Goree LL, Chandler-Laney PC, Ellis AC, Casazza K, Granger WM (2012). A higher-carbohydrate, lower-fat diet reduces fasting glucose concentration and improves β-cell function in individuals with impaired fasting glucose. Metabolism.

[25] Chen C, Yu X, Shao S (2015). Effects of omega-3 fatty acid supplementation on glucose control and lipid levels in type 2 diabetes: A meta-analysis. PLoS One.

[26] García-Conesa MT, Philippou E, Pafilas C, Massaro M, Quarta S, Andrade V, Jorge R, Chervenkov M, Ivanova T, Dimitrova D (2020). Exploring the validity of the 14-Item Mediterranean Diet Adherence Screener (MEDAS): A cross-national study in seven European countries around the Mediterranean region. Nutrients.

[27] Yan S, Li M, Ma X, Jiang S, Sun M, Wang C, Pan Y, Sun C, Yao Y, Jin L, Li B (2019). Association of multiple mineral and vitamin B group intake with blood glucose using quantile regression analysis: NHANES 2007–2014. Food Nutr. Res..

[28] Feart C, Alles B, Merle B, Samieri C, Barberger-Gateau P (2012). Adherence to a Mediterranean diet and energy, macro-, and micronutrient intakes in older persons. J. Physiol. Biochem..

[29] Tsai HJ (2016). Dietary patterns and depressive symptoms in a Taiwanese population aged 53 years and over: Results from the Taiwan Longitudinal Study of Aging. Geriatr. Gerontol. Int..

[30] Leri M, Scuto M, Ontario ML, Calabrese V, Calabrese EJ, Bucciantini M, Stefani M (2020). Healthy effects of plant polyphenols: Molecular mechanisms. Int. J. Mol. Sci..

[31] Tuttolomondo A, Simonetta I, Daidone M, Mogavero A, Ortello A, Pinto A (2019). Metabolic and vascular effect of the Mediterranean diet. Int. J. Mol. Sci..

