# Supplementary material for: Short Mediterranean diet screener detects risk of prediabetes in Taiwan, a cross-sectional study
Source: Sci Rep. 2023 Jan 21;13:1220. doi: 10.1038/s41598-023-28573-5 (PMC9867715; doi:10.1038/s41598-023-28573-5)
Supplement: Supplementary file 1 — Supplementary Information. [file 41598_2023_28573_MOESM1_ESM.docx]

1. KMO(Kaiser-Meyer-Olkin) and Bartlett’s test

The KMO value of the “Mediterranean Diet” scale is 700, and the KMO value of the scale reaches .70, which belongs to the middling standard, while the Bartlett’s test value of sphericity reaches a significant level (p < .001), showing that the scale’s Data suitable for performing factor analysis.

KMO(Kaiser-Meyer-Olkin) and Bartlett’s test result of MEDAS

|  | | MEDAS |
| --- | --- | --- |
| Kaiser-Meyer-Olkin measure of sampling adequacy | | .700 |
| Bartlett test of sphericity | χ^2^ | 639.984*** |
|  | *df* | 91 |
|  | *p* | <.001 |

****p*<.001

1. Reality analysis

According to the Cronbach’s α coefficient of the deleted items, this research deletes the items in order and updates the α coefficient as shown below.

When undertaking the factor analysis results and only deleting the fifth question for reliability analysis, the Cronbach’s α coefficient .615 does not reach the standard of .70, after the 11^th^ and 13^th^ questions must be deleted, the Cronbach’s α coefficient of the Mediterranean Diet Scale is .707, which meets the standard of good reliability. At this time, there are 10 items left.

| process | Number of question | Cronbach’s α coefficient |
| --- | --- | --- |
| Undertake factor analusis results | 13 | .615 |
| Delete Q11 | 12 | .667 |
| Delete Q13 | 11 | .686 |
| Delete Q6 | 10 | .707 |
| Delete Q8 | 9 | .723 |
| Delete Q7 | 8 | .726 |
| Delete Q3 | 7 | .727 |
| Delete Q4 | 6 | .730 |
| Delete Q10 | 5 | .731 |

**Supplementary Table S2.-** Agreement between the FFQ-MEDAS and the 3d-FD: per-item validation analysis (κappa statistics) in the whole sample population (all countries).

| Question | Score | 3d-FD  (% scoring 1) | FFQ-MEDAS^1^  (% scoring 1) | % Absolute agreement | κ (95%CI)  (3d-FD *vs* FFQ-MEDAS(1) |
| --- | --- | --- | --- | --- | --- |
| 1.- Olive oil | yes | 2.0 | 21.0 | 81.0 | 0.143 (-0.036 - 0.321) |
| 2.- Olive oil | ≥4 | 0.0 | 2.0 | 98.0 | NA |
| 3.- Vegetables | ≥2 | 9.0 | 14.0 | 91.0 | 0.561 (0.307 - 0.814) |
| 4.- Fresh fruits | ≥3 | 7.0 | 12.0 | 93.0 | 0.596 (0.328 - 0.864) |
| 5.- Red & processed meat | <1 | 71.0 | 76.0 | 89.0 | 0.719 (0.564 - 0.873) |
| 6.- Butter, margarine | <1 | 98.0 | 96.0 | 98.0 | 0.658 (0.216 - 1.099) |
| 7.- Sweet beverages | <1 | 65.0 | 81.0 | 84.0 | 0.607 (0.445 - 0.769) |
| 8.- Wine | 7 to14 | 1.0 | 7.0 | 94.0 | 0.237 (-0.146 - 0.619) |
| 9.- Legumes | ≥3 | 25.0 | 36.0 | 85.0 | 0.651 (0.495 - 0.808) |
| 10.- Fish & seafood | ≥3 | 11.0 | 11.0 | 94.0 | 0.694 (0.463 - 0.924) |
| 11.- Desserts | <3 | 62.0 | 61.0 | 89.0 | 0.768 (0.638 - 0.897) |
| 12.- Nuts | ≥3 | 9.0 | 19.0 | 90.0 | 0.593 (0.375 - 0.812) |
| 13.- White over red meat^2^ | ≤1 or yes | 46.0 | 39.0 | 71.0 | 0.410 (0.231 - 0.588) |
| 14.- ‘Sofrito’ | ≥2 | 12.0 | 30.0 | 78.0 | 0.368 (0.175 - 0.560) |
| Mean value |  | 29.9 | 21.0 | 88.2 |  |

^1^: Mean value of FFQ-MEDAS (1) and FFQ-MEDAS (2); ^2^: ≤1 for the 3d-FD and 'yes' for the FFQ-MEDAS; ^3^ к ≤ 0 no agreement (small negative values) or disagreement (large negative values), к = 0.01 − 0.20 slight, к = 0.21 − 0.40 fair, к = 0.41 − 0.60 moderate, к = 0.61 − 0.80 substantial, к = 0.81 – 1.0 almost perfect [26].
